# Commentary: From little things big things grow

**DOI:** 10.1016/j.xjtc.2021.10.054

**Published:** 2021-10-29

**Authors:** Yaroslav Ivanov, Edward Buratto, Antonia Schulz, Igor E. Konstantinov

**Affiliations:** a Department of Cardiac Surgery, Royal Children's Hospital, Melbourne, Australia; b Department of Paediatrics, University of Melbourne, Melbourne, Australia; c Heart Research Group, Murdoch Children's Research Institute, Melbourne, Australia; d Melbourne Centre for Cardiovascular Genomics and Regenerative Medicine, Melbourne, Australia

**Figure:**
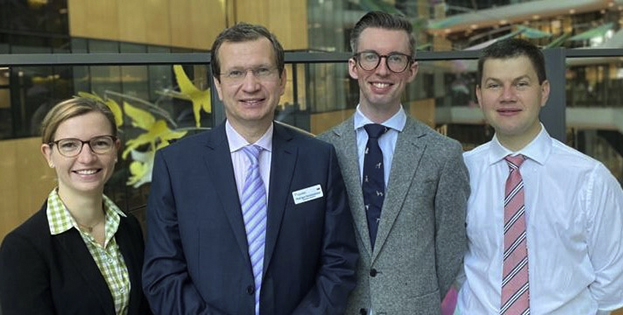
Antonia Schulz, MD, Igor E. Konstantinov, MD, PhD, FRACS, Edward Buratto, MBBS, PhD, FRACS, and Yaroslav Ivanov, MD, PhD

**Central Message**

Inferior vena cava stenosis following transvenous leads placement, although avoidable, may require a complex surgical approach.

See Article page 31.

The rate of implantation of the transvenous automated implantable cardioverter-defibrillators (AICDs) and pacemakers is ever increasing due to the relative simplicity of the implantation and high efficacy.^1^ However, percutaneous implantable devices are not without their shortcomings.^1^, ^2^, ^3^, ^4^ As their delivery route is the superior vena cava (SVC) system, not surprisingly most of the complications occur within the deep veins of the upper extremity.^2^ Both venous thrombosis^1^ and stenosis may occur, leading to occlusion.^2^ Most strikingly, the overall prevalence of asymptomatic vein occlusion from a recent meta-analysis study is 8.6%.^1^ Interestingly, even with complete occlusion of SVC, patients may remain free of symptoms, suggesting that a gradual occlusion may allow collaterals to develop.

In the current issue of the *Journal*, Smith and colleagues^5^ described a patient with complete occlusion of SVC and severely stenotic inferior vena cava (IVC) many years after the AICD implantation. The patient developed the portal hypertension and ascites. The almost-complete occlusion of IVC was treated surgically with IVC patching after other therapeutic and endovascular variants were exhausted. Interestingly, even with complete removal of the AICD system the SVC occlusion remained, yet the patient had complete resolution of the symptoms due to well-developed collaterals. Surgical management of cardiac lesions located at the right atrium to IVC junction is not always straightforward.^6^ Ironically, this avoidable complication could have been easily prevented by proper placement of the transvenous leads. The best way to deal with complications is to prevent them!
